# Willingness to Pay for Enhanced Mandatory Labelling of Genetically Modified Soybean Oil: Evidence from a Choice Experiment in China

**DOI:** 10.3390/foods10040736

**Published:** 2021-03-30

**Authors:** Mingyang Zhang, Yubing Fan, Jingxia Cao, Lijun Chen, Chao Chen

**Affiliations:** 1 School of Business, Development Institute of Jiangbei New Area, Nanjing University of Information Science and Technology, 219 Niuliu Road, Nanjing 210044, China; 002668@nuist.edu.cn (M.Z.); 15162780938@163.com (J.C.); 2 Texas A&M AgriLife Research, 11708 Highway 70 South, Vernon, TX 76384, USA; yubing.fan@ag.tamu.edu; 3 Food and Resource Economics Department, University of Florida, McCarty B 2115, Gainesville, FL 32611, USA; chenlij@missouri.edu; 4 College of Economics & Management, Nanjing Agricultural University, 1 Weigang, Nanjing 210095, China

**Keywords:** GM foods, food labelling, soybean oil, willingness to pay, choice experiment

## Abstract

This study investigates consumers’ preferences for mandatory labelling conveying the health and safety attributes of genetically modified soybean oil. The enhanced mandatory labelling includes allergen presence labelling, nutrient and compositional change labelling and traceability codes. The data were collected from a consumer survey in the eastern, central and western regions of China, with a total sample size of 804 respondents. We evaluated consumer willingness to pay (WTP) for enhanced mandatory labelling using a choice experiment approach. The results show that Chinese consumers are most favorable to traceability codes with a WTP of RMB 8.92, followed by allergen presences labelling, with RMB 6.57. Eastern consumers would like to pay a higher premium for the three types of enhanced mandatory labelling information, while central consumers only show a positive preference for traceability codes. The results imply that the efforts and policy strategies for enhanced mandatory labelling will benefit residents. Further studies can be expended to other genetically modified (GM) foods. This study provides information for the agency to improve mandatory GM food labelling management. This paper contributes to the growing body of the GM food literature by explicitly investigating consumer preference and WTP for mandatory labelling conveying the health and safety attributes of the GM foods.

## 1. Introduction

As a fast developing country, China is the largest consumer and importer of genetically modified (GM) soybean [1]. The import of soybeans to China reached more than 95 million tons in 2017, which were used as processing materials [2]. Chinese consumers oppose GM foods due to their concerns about food safety [3]. Concerns of the potential risks of the GM foods are growing, particularly among civil society groups [4]. The lack of effective communication on GM foods leads to asymmetric information [5,6]. Mandatory labelling is considered a practical way to address the issue of asymmetric information in food labelling [7]. In order to disclose foods that are or may be bioengineered, the National Bioengineered Food Disclosure Law of U.S., passed by Congress in July 2016, directed the U.S. Department of Agriculture to establish the national mandatory standard. The National Bioengineered Food Disclosure Standard, which was announced in 2018, is simply a marketing label, and does not convey any information about the health, safety, or environmental attributes of the GM foods in comparison with their non-GM counterparts [8]. Similarly, China currently adopts qualitative mandatory labeling according to the catalogues, and the labelling catalogue includes 17 kinds of GM products within five categories, including soybeans, rape, corn, cotton, and tomato. As one of the directly processed GM agricultural products, the label of the GM soybean oil merely tells customers “the raw materials are GM soybeans.” The usefulness of the simple GM food label is limited for consumers, because the current labelling only allows differentiating GM foods from non-GM food products [9]. Better information could increase consumers’ support for GM foods, which further determines the implementation and success of the mandatory labelling policy [10]. However, mandatory labelling conveying the health and safety attributes for the improved transparency and openness of information inevitably leads to an increase in production cost, such as detecting cost. Therefore, this study investigates consumers’ preferences for mandatory labelling conveying the health and safety attributes of the GM soybean oil. 

Focusing on the developed countries, i.e., the US and European countries, much research has investigated consumer attitudes towards the mandatory labelling of the GM foods [11,12]. A research consensus has been reached that consumers may have greater demands for the mandatory labelling of the GM foods [11,12,13,14]. However, there are limited systematic studies on consumers’ willingness to pay for the extra cost associated with the mandatory labelling information, especially on consumer preference and willingness to pay (WTP) for mandatory labelling conveying health and safety attributes. To the best of the authors’ knowledge, no empirical study has been reported on WTP for the mandatory labelling of the GM foods in China.

Given the importance of mandatory labelling, this paper contributes to the literature by empirically analyzing urban consumers’ preferences for mandatory labelling conveying health and safety attributes. Additional insights are provided for public service by evaluating consumers’ WTP for the enhanced labelling of the GM soybean oil, including allergen presence labelling, nutrient and compositional change labelling, and traceability codes.

## 2. Literature Review

### 2.1. Debate on GM Food Labelling

Academia is divided on the pros and cons of the GM food labelling. On the one hand, some scholars holding a positive view believe that labelling can effectively tackle the problem of asymmetric information, which is of great benefit to forming a market with a remarkable separation of the GM and non-GM foods [14,15]. Mandatory labelling also contributes to GM food management, highlighting information such as place of origin, allergen presence, and detailed food ingredients, which are conducive to government regulation. Especially, the government can take timely and effective actions if any GM food safety problems occur [16,17]. The labelling policy has been found to be superior to an embargo in terms of consumer welfare and producer benefits [18]. In addition, GM labelling is closely related to consumers’ right-to-know and assists consumers in making better informed purchase decisions [5].

On the other hand, other scholars holding a negative view believe that mandatory labelling obviously adds extra costs to the production and society, such as the adjustment cost, implementation cost, and monitoring cost [15,17]. For instance, upon the introduction of mandatory labelling in Europe and US, production costs increased by 17% and 6%, respectively [19]. If the GM food labels were added, each US household would pay an estimated USD 100 more on food every year [20].

### 2.2. Consumer Attitude towards GM Labelling

Relevant studies on consumer attitudes towards mandatory GM food labelling have reported findings in both developed and developing countries. In general, consumers have a strong preference for mandatory GM food labelling [11]. Marchant and Cardineau [21] analyzed the labelling debates in the US. Public opinion polls consistently show that 90 percent or more of Americans want foods to be labeled. Luck et al. [22] reported that over 80 percent of American consumers are supportive of implementing the mandatory labelling policy on GM products. Nep and O’Doherty [14] used data from a deliberative public engagement in British Columbia of Canada. In their survey, participants discussed the social and ethical implications of salmon genomics. The public called for mandatory labelling of transgenic salmon, and demanded labelling as a minimum requirement to allow consumers to choose whether to purchase GM foods. Participants showed strong distrust in the current supervision of the GM foods, and the perceived reluctance of biotechnology companies serves to fuel this distrust. 

Further, much research has been conducted in developing countries. Huang and others [23] investigated 400 participants in Wuhan, China, and they found that more than four-fifths Chinese consumers demanded implementing mandatory GM food labelling policies. Deng et al. [24] found more than 90 percent of participants supported mandatory labelling, based on a survey of 260 participants from 11 provinces in China. Zhao et al. [25] investigated 1730 Chinese respondents’ attitudes toward five different GM food labelling methods including no GM label, labels of meat fed by GM feeds, labels of cooking oil containing GM oil, labels of the GM condiments, and labels of non-GM ingredients. They found that those who were more familiar with genetically modified organisms (GMOs) or who trusted the government were more positive about GM labels. Kajale and Becker [13] conducted an interview among a sample of 298 students in India. They found that about 58 percent of college students supported mandatory GM food labelling, and about 44.63 percent believed the increased price should be jointly paid by consumer, producer and government.

### 2.3. Information Credibility and Adequacy of the GM Food Labels

U.S. consumers desire GM food labels to provide sufficient information relating to potential benefits and risks, which implies that the usefulness of a simple GM food label is limited for the public. As simple labels just allow consumers to differentiate GM food products from their non-GM counterparts, they do not include enough of the benefit and risk information that consumers desire to know [9]. Teisl and others [9] indicated that a simple GM label actually may not be beneficial to consumers who are anxious about GM contents but may be willing to accept the GM foods if the genetic modification provides any benefits. Moreover, excessive information on a GM food label may negatively affect consumers if they have limited knowledge of genetic engineering and GM foods [26]. Roe and Teisl [27] presented US consumers with some sample labels that contained different statements concerning the presence of the GM ingredients, and the consumers evaluated the credibility and adequacy of the information content. The result showed that a simple GM label just saying a product contains GM ingredients was considered more credible than the simple non-GM labels saying a product contains no GM ingredients. However, the consumers were more likely to judge the simple non-GM label as having provided an adequate amount of information for informed decisions to be made. They also found several significant improvements in the adequacy of simple GM labels when they mentioned the purpose of the GM usage, which significantly eroded the label’s credibility rating. Hence, label credibility and label adequacy may remain opposite, but the provision of contact information may help resolve the credibility–adequacy trade-off.

### 2.4. Consumers’ WTP for GM-Labeled Foods

Wolfe and others [11] found a significant premium for non-GM edamame even if there is no obvious difference between the overall sensory impression of the GM edamame and the non-GM counterparts in the US. This finding was similar to Huffman et al. [28] where a 14% premium was reported for non-GM vegetable oil, tortilla chips and potatoes compared to the GM-labeled counterparts. Likewise, Lusk et al. [29] found a premium of 25 cents per ounce for non-GM corn chips. Other scholars also reported similar findings and explained WTP for several kinds of the GM foods based on various functional GM foods (i.e., yield increasing, ripening controlling, protective, processed, nutrition improving GM foods), and crop classification (i.e., GM rice, GM vegetables, GM fruit, GM edible oil, etc.) [30,31,32,33]. However, there are limited systematic studies on the WTP for the extra cost associated with the mandatory labelling information, especially for the preference and WTP for mandatory labelling conveying health and safety attributes. In particular, no empirical research has been reported in China.

## 3. Choice Experiment

### 3.1. Identifying GM Labelling Policies

Currently, the GM organism labelling policies around the world fall into two types. One type is voluntary labelling, such as in Canada; the other is mandatory labelling, such as in the US, the EU, and China. In order to determine the exact rules applicable to labelling in the international and national context, we analyze the compilation of the codex committee on food labelling (CCFL), Canada’s labelling policies, and the labelling legislation in the EU and its implementation in England. Since 1993, CCFL has begun to discuss the issue of the GM food labelling (1997 Text, 2001 Text, 2004 Text, 2008 Text, 2009 Text, 2010 Text and 2011 Text). Although it has yet to form a generally accepted international standard, the above text in the mandatory labelling has reached a consensus: there must be mandatory labelling in the presence of allergens [34]. Nevertheless, neither the regulations regarding the mandatory labelling of the GM food nor the provisions relating to the thresholds, exemptions and implementation are the same. The voluntary labelling model adopted in Canada requires labelling in the event of the presence of allergens or changes in the nutritional value or components [35]. The EU traceability and labelling regulation 1830/2003296 seeks to address the concerns about the lack of information to enable the labelling of the GM foods, and sets out the requirements for a document audit trail to account for and identify approved GM products throughout the marketing chain. This regulation summarizes the purpose: the traceability requirements for food and feed produced from GMOs should be established to facilitate the accurate labelling of such products. Its objective is to enable postmarket monitoring of health and the environment [35].

### 3.2. Experimental Design

A choice experiment (CE) approach was used to evaluate urban consumers’ WTP for the attributes of enhanced mandatory labelling of the GM soybean oil. The CE model relies on random utility theory and factor value theory, and they indicate that the utility is from the attributes possessed by the item rather than item itself [36]. As for the enhanced mandatory GM food labelling, the combination of the labelling attributes and choice scenarios are formulated in the CE. Specifically, the consumer can obtain the utility $v_{k}$ from the *k-th* labelling attribute, and the utility *V*, obtained from enhanced mandatory GM food labelling, equals to the sum of the utility $v_{k}$ (k = 1, 2, …, K).

(1) $$V=\lambda_{1}v_{1}+\lambda_{2}v_{2}+\ldots +\lambda_{k}v_{k}$$

where $\lambda_{k}$ is the unknown parameter, referring to individual’s preference for utility $v_{k}$. Consumer *i* must evaluate the utility $U_{imn}$ from the enhanced mandatory GM food labelling associated with the alternative m=1,2,…,M in the *n-th* choice set. Within a given group of alternatives relating to a choice set, the consumer selects the utility-maximizing alternative. $U_{imn}$ is a random variable that can be expressed as:

(2) $$U_{imn}=V_{imn}\alpha +\mu_{mn}$$

where $\alpha_{mn}$ refers to the estimated parameter vector. $\mu_{mn}$ is the random disturbance term. The vector $V_{imn}$ means sum of the utility obtained from the mandatory labelling attribute and payment vehicle associated with the alternative m=1,2,…,M in the *n-th* choice set [36].

This study adopts a choice experiment model on the GM soybean oil sales and labelling in China Including Regulations on Administration of Agricultural Genetically Modified Organisms Safety and Administrative Measures for Agricultural GMOs Labeling issued by the Ministry of Agriculture and Rural Affairs of P. R. China. This study follows the relevant literature [26,27,35,37,38,39,40], and the representative GM organism safety management policies. The CE model contained three labelling attributes and the payment vehicle (Table 1), that is, allergen presence labelling, nutrient and compositional change labelling, traceability codes, and price.

**Figure 1:**
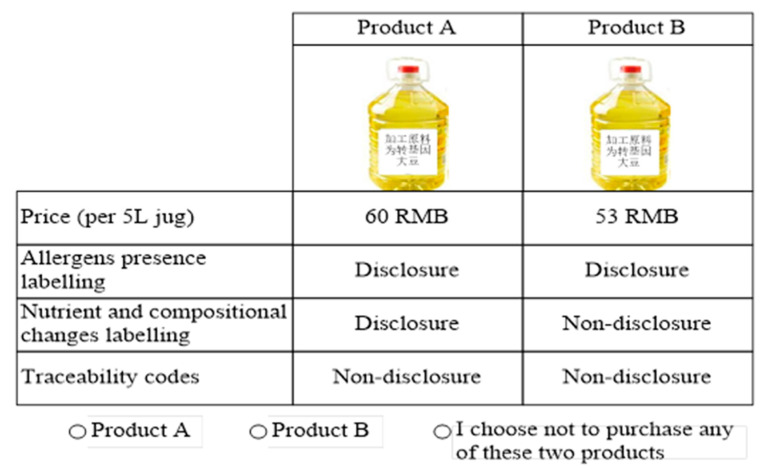
Example choice scenario.

Each of the first three attributes include two levels (disclosure or nondisclosure), and the price includes three levels. Thus, there were 24 possible combinations in total. We can constitute 276 CE scenarios by pairing those combinations. After eliminating both the overlapping and theoretically contradictory CE scenarios, we conduct the screening experiment, and obtain twelve CE scenarios. These scenarios are randomly divided into two groups, with each contain six CE scenarios. A sample CE scenario is shown in (Figure 1).

In the experiment, GM soybean oil is selected as the analysis unit for four reasons. Firstly, the studies showed that Chinese consumers preferred to accept foods derived from bioengineered food rather than directly edible GM foods like GM soybean oil [41]. Therefore, it can be inferred that consumer demand for labelling information for the directly edible GM foods is the most urgent. Secondly, according to the GM organism safety certificates for both commercial planting and GM organisms imported as raw materials approved by the Ministry of Agriculture and Rural Affairs of China, currently there are only three kinds of directly edible GM foods on the Chinese market; that is, locally grown GM papaya, GM soybean oil and GM canola oil made from imported GM soybeans or rapeseed. The GM oil is labeled “The processing material is GM soybeans or rapeseeds.” Thirdly, soybean oil is not only the most daily consumption edible oil in the majority of Chinese cities, but also the most popular with food processing enterprises and the catering industry. Fourthly, there are a wide range of alternatives to GM soybean oil and non-GM soybean oil available on the Chinese market, such as non-GM peanut oil, non-GM corn oil, non-GM sunflower oil, non-GM canola oil, non-GM rapeseed oil, and many kinds of oil blends. There are a variety of brands of edible oil in the Chinese market. Some only sell non-GM soybean oil (such as the Northeast soybean oil, Xinheshun and Qiansuihao, etc.), while some only sell GM soybean oil (such as Fortune, Jinlongyu, YuanBao, Fivelakes, etc.). Most enterprises produce only one or a few of the edible oils (such as only producing soybean oil, peanut oil, corn oil, sunflower oil, olive oil, rapeseed oil, or blended oil). In order to ensure the GM soybean oil, non-GM soybean oil and oil blends are identical in the brand, capacity and other aspects, this section uses “X” brand edible oil as the experimental unit, which is one of the top ten well-known brands of edible oil in China. Except for peanut oil, all other soybean oil substitutes are supplied in a 5-liter jug. 

The prices of all kinds of “X” brand 5-liter edible oil are shown in (Table S1), which was presented to the respondents in the experiment. In this study, the price of 5L GM soybean oil (RMB 45.8, 1 USD=6.80 RMB) is set as the lower limit, and the price of 5L non-GM soybean oil (RMB 66.8) is set as the upper limit. According to the principle of isometric and rounding, the price is set at three levels: RMB 46, RMB 53, and RMB 60.

Additionally, the CE model follows a “randomized design” developed by Sawtooth Software, Inc. [42]. Compared to the fixed design, the randomized design can eliminate order and psychological context effects [43]. Additionally, the randomized designs are more efficient in asymmetric choice experiments when not all attributes have equal levels [44].

### 3.3. Mixed Logit Model

The mixed logit (ML) model is used to analyze the data collected in the choice experiment. The ML model (specified in the Equation (3) below) relaxes the independent of irrelevant alternative (IIA) assumption and allows individual variations in the attributes [45]. Meanwhile, the conditional logit model is typically used if the random terms follow independently identically distribution (IID) and assumes respondents having the same preference for the attributes. The likelihood ratio (LR) tests can be used to compare the two models [46]. If the null hypothesis that there is no difference between the two models is rejected, this indicates the ML model is more appropriate. In addition, a probability density function, g(α), is introduced for the coefficient of the presumed heterogeneous attributes. Namely, correlations between preferences are allowed and different respondents show different preferences for the attributes of enhanced mandatory labelling. The non-conditional probability $P_{imn}$ of consumer *i* who chooses the *m*-*th* alternative in the *n-th* choice scenario can be get by calculating the integral of g(α) with respect to α.

(3) $$P_{imn}=\int \frac{\exp\left(V_{imn}\alpha \right)}{\sum_{m=1}^{M}\exp\left(V_{imn}\alpha \right)}$$

The model assumes that g(α) functions of all nonpayment attributes follow normal distributions. The price attribute with a fixed coefficient equals the given market price and the other two reference prices are slightly higher than the market price. The parameter α refers to scaled marginal utility for a mandatory labeling attribute or price, due to scale normalization. Therefore, we can only interpret the relative magnitude of the other attributes and statistical significance by the parameter estimates. The WTP can be calculated from the negative marginal utility divided by the coefficient ($\alpha_{p}$) of the price attribute [47]. Therefore, they are comparable across the results.

(4) $$WTP=-\frac{\alpha_{k}}{\alpha_{p}}$$

## 4. Data

### 4.1. Survey Administration

The survey contained questions designed for the experiment and socio-demographic inquiries including gender, age, educational attainment, occupation, child, and income. The respondents were first provided with some detailed information on GM soybean imports and their connection to public interest. Each attribute was interpreted by the enumerators to make sure the respondents understood the survey. All the nonpayment attributes and price attributes in (Table 1) were shown to the respondents, who were asked whether they were willing to pay for the mandatory enhanced labelling of the GM soybean oil. They were also shown a sample of a CE scenario (see Figure 1) before the start of the experiment. We showed them what it would mean if “Product A” was chosen. The prices of all kinds of “X” brand 5-liter edible oil are shown in (Table S1), which were also presented to the respondents in the experiment. Two versions were developed to reflect the differences in the CE scenarios. Each respondent only took one version of the survey, assigned to six choice scenarios.

Adhering to the stratified random sampling, a self-administered questionnaire was utilized to collect data in the provincial capitals of the eastern, central and western regions. The eastern regions include Jinan of Shandong province, Nanjing of Jiangsu province, Shanghai, and Guangzhou of Guangdong province. The central regions include Changchun of Jilin province, Zhengzhou of Henan province, Hefei of Anhui province, Wuchang of Jiangxi province. The western regions include Lanzhou of Gansu province, and Guiyang of Guizhou province. This investigation was conducted at supermarkets and large-scale shopping malls by sixty-four undergraduates from Nanjing Agricultural University in 2017. Different social classes were sampled to avoid sample selection bias owing to sampling at a single site [48]. The survey enumerators approached potential respondents and invited them to participate if they wanted. The following steps were followed: (1) each respondent was confirmed to be an urban resident; (2) the selected respondents had food purchasing experience; (3) soybean oil was the family’s main edible oil and was obtained through purchasing rather than through squeezing their own beans. After completing the survey, each respondent was offered a RMB 10 gift.

### 4.2. Descriptive Statistics

This analysis is based on 804 samples collected in the survey. The samples in the eastern, central and western regions account for 45.02%, 35.07% and 19.90%, respectively. Specially, there are 2172, 1688, and 960 choice scenarios in the eastern, central and western regions, respectively, because each respondent responds to six choice scenarios. A statistic summary of socio-demographics of the sampled urban consumers is shown in (Table 2). Compared with the population, i.e., the national urban and rural residents, from the 2017 China statistical yearbook, the sample includes fewer males and shows a better education attainment, with 63.18% attending a professional school or holding a college or higher degree. The sample includes more young people under 45 years old. In addition, about 8% of the respondents have a job relating to biotechnology, and 56.47% of the families have minors. The average monthly household disposable income roughly follows a normal distribution, with the categories RMB 4001–6000, RMB 6001–8000, and RMB 8001–10,000 accounting for 31.84%, 16.92%, and 12.69%, respectively. 

Table 3 shows a statistical summary of the variables used in the ML regressions. The consumers who are willing to pay for the enhanced mandatory labelling of the GM soybean oil account for 67.40%, 58.90%, and 57.50% in the eastern, central and western regions, respectively. From the nationwide perspective, the means of the three attributes are −0.087, −0.044, and −0.011, respectively. The average of the prices is RMB 35.21.

## 5. Estimation Results

We conducted the mixed logit regressions using the simulated maximum likelihood estimator. Firstly, the correlation test shows that there is correlation between each pair of the attribute variables. For example, the correlation coefficient between allergen presence labelling and nutrient and compositional change labelling is 0.74. Obviously, the correlation test result is in conflict with the IID assumption of the conditional logit model. Therefore, we run the ML model with correlated normally distributed coefficients. The result of the LR test for the nationwide sample is 3858 and significant at *p* < 0.001, which indicates that the null hypothesis is rejected. In other words, the respondents have heterogeneous preferences. Therefore, the conditional logit model has a poorer fit compared to the ML model.

Table 4 provides the estimation results of respondents’ preferences for the enhanced mandatory labelling of the GM soybean oil. The results show that price has a negative effect in all equations, indicating an increase in GM soybean oil price decreases the probability of a consumer choosing the oil. More importantly, most proposed enhanced GM mandatory labelling information is positive.

The coefficients of the three variables of labelling attributes are 0.427 (*p* < 0.01), 0.104, and 0.579 (*p* < 0.01) on a national scale (Table 4). The results suggest that Chinese urban consumers are in favor of the enhanced mandatory labelling of the GM soybean oil. The most attractive and influential labelling attribute is traceability codes, followed by allergen presence labelling with a smaller effect. The least important labelling is nutrient and compositional change labelling, and consumers are more likely to select an alternative based on other enhanced labelling included.

To better understand consumers’ preference for mandatory labelling conveying health and safety attributes, we estimate the WTP values using the parameter estimates from the ML model. The WTP values for each attribute are shown in Table 5. The magnitude of WTP and their ranks are consistent with that of the coefficient estimates from the ML model (Table 4). Positive WTP values represent the amount of money that the consumers are willing to pay for the specific labelling attributes. The highest WTP value is found for the traceability codes. Specifically, the consumers are more likely to pay for the traceability codes nationwide, with a payment of 8.92 RMB, followed by the allergen presence labelling with a value of 6.57 RMB. Regionally, eastern consumers show a positive preference for all three attributes, with the payment amounts of 11.24 RMB, 8.87 RMB, and 6.48 RMB for traceability codes, allergen presence labelling, and nutrient and compositional change labelling, respectively. Central consumers only show a positive preference for the traceability codes, i.e., 7.41 RMB. However, western consumers show no preference.

Furthermore, the survey results show that about 62.44% of the urban consumers state that they are willing to pay for the enhanced mandatory labelling (Table 3). For those who are willing to pay, the average WTP is RMB 18.22 for traceability codes, followed by RMB 17.50 for allergen presence labelling. The WTP for nutrient and compositional change labelling is the smallest with a payment amount of RMB 8.17. 

## 6. Discussion

Chinese urban consumers show a positive preference for mandatory labelling conveying some information about the safety attributes of GM foods. This is largely because a simple GM food label, such as “the raw material is GM soybeans,” only allows differentiating GM foods from their non-GM counterparts. Most consumers would like to see more detailed information about the potential benefits and risks on GM food labels [9]. Urban consumers are more likely to pay for traceability codes, followed by allergen presence labelling, while nutrient and compositional change labelling is least important. Our results are consistent with the findings of Roe and Teisl [27] who suggested that providing contact sources that consumers can use to obtain more information could resolve the credibility–adequacy trade-off. They also proposed several improvements in the adequacy of simple GM labels, such as adding the purpose of the GM ingredients’ usage. While this addition on the label also greatly erodes the label’s credibility and retains the opposition of label credibility and adequacy [27].

Our results show moderate regional heterogeneity in the preference and WTP. Among those who are willing to pay, the western consumers show a strong preference for allergen presence labelling and traceability codes with the values of RMB 17.58 and RMB 24.76, respectively, while central consumers also have a stronger preference for both allergen presence labelling and traceability codes, and the WTP values are RMB 18.28 and RMB 18.57, which are higher than the eastern levels. On average, the per capita disposable income in the eastern cities is higher than that in the central cities, which is turn higher than that in the western cities [49]. Meanwhile, the consumers in the higher income region would not like to pay a premium for the enhanced mandatory labelling of the GM soybean oil. Additionally, we found that compared to the respondents who are unwilling to pay, the respondents who are willing to pay have a lower per capita disposable income. This is consistent with the findings of Wolfe and others [11] who reported that the urban households with a higher income can afford the non-GM oil. In general, the higher income households are more cautious about food choice [50,51]. Thus, they are more likely to purchase the non-GM oil rather than being willing to pay for the enhanced labelling of the GM foods. For lower income consumers, GM oil may be their main edible oil because of the low price. They would like to know more about GM foods, and have a stronger demand for right-to-know. Mandatory labelling may be a practical way to address the issue of asymmetric information in food labelling [7]. Hence, consumers from western China would like to pay a higher premium for the enhanced mandatory labelling of the GM soybean oil. 

On average, the eastern consumers have a higher education level than those from the central region, whose education level is in turn higher than that of consumers from the western region. Educational attainment may determine food preference; therein lies a useful pointer for the policy makers [52]. Well-educated consumers may be more concerned about GM foods, because they may be worried about the uncertainty of transgenic technology, but they may be not in fact be aware of GM products [53,54]. 

## 7. Conclusions

This paper contributes to the growing body of the GM food literature by explicitly investigating consumer preference and WTP for mandatory labelling conveying the health and safety attributes of the GM foods. The results signify that consumers recognize the importance of investing in the mandatory labelling conveying safety information. This suggests the efforts and policy strategies for enhanced mandatory labelling will benefit Chinese citizens. This is encouraging because financial and technical assistance from the government can target certain interest groups, rather than distributing the resources to satisfy all groups. It may be more interesting to agency leaders to consider the specific WTP amounts for the three types of enhanced mandatory labelling information. Allergen presence labelling and nutrient and compositional change labelling can better help consumers understand the potential risks and benefits of the GM foods, but neither is highly ranked in terms of the WTP values. Instead, the traceability codes show the highest WTP value. This is interesting because the traceability codes may help consumers know where the products come from, but it would not inform them of the potential risks and benefits. Conversely, it may be the nature of the right-to-know of traceability codes that makes them more valuable to the public. While government agencies are responsible for improving mandatory GM food labelling management for the benefit of the public, it is critical to include publicly linked policies, such as consumer WTP for enhanced labelling, to gain more support from the public. 

Policies encouraging consumers to make purchase decisions that match personal preference are inherently desirable, regardless of the end-user characteristics or process attributes. These policies should be cost-effective. Unfortunately, our results do not present the costs or benefits of instituting an enhanced mandatory labelling program. A policy decision to impose enhanced mandatory labelling should recognize both its benefits and costs, while considering whether the practitioners are equipped or facilitated to implement the policy. The research does not conclude that an enhanced mandatory labelling program should be instituted. Rather, the findings provide guidance on how an enhanced mandatory labelling program should look like if such a program is warranted. Nevertheless, further research is needed, including calculating the additional costs and evaluating the benefits. Additionally, this paper is restricted to GM soybean oil, while future research can expand this approach to other GM foods.

## Figures and Tables

**Table 1:** Attributes and levels in the choice experiment.

Attributes	Levels	Description	Basis
(1). Allergens presence labelling	Disclosure, nondisclosure	Disclose the presence of allergens in GM soybeans oil	a, b, c
(2). Nutrient and compositional change labelling	Disclosure, nondisclosure	Disclose the changes in nutritional value or composition of the GM soybean oil, compared with non-GM counterpart.	b, c
(3). Traceability codes	Disclosure, nondisclosure	Traceability systems document the entire process of the GM soybean oil production. The systems allow for the separation of the GM soybean oil and non-GM products “from farm to fork,” and serve the purpose of marketing and health protection.	d, e
(4). Price (RMB)	46, 53, 60	Price of a 5L jug of the GM soybean oil.	

a: Seven texts drafted by the Codex Alimentarius Commission (CAC) on Food Identification Subcommittee in the past decades. b: Food and Drugs Act, Food and Drug Regulations, and Consumer Packaging and Labeling Act by Canada; c: Draft Guidance for Industry: Voluntary Labeling Indicating Whether Foods Have or Have Not Been Developed Using Bioengineering by US; d: Traceability and Labelling Regulation and Regulation (EC) on Novel Foods and Novel Food Ingredients issued by EU; e: Genetically Modified Organisms (Traceability and Labelling) (England) Regulation 2004.312; RMB 6.80 = USD 1.

**Table 2:** Definition and descriptive statistics of the demographic variables.

Variable	Description	Sample Mean ^a^	Population Mean ^b^
Gender	0 = male; 1 = female.	0.500 (0.500)	0.499
Age	0 = young people (18–44); 1 = middle-aged or senior people (≥45).	0.254 (0.435)	
Education attainment	0 = senior high school or below; 1 = professional school, college degree or above.	0.632 (0.483)	0.401
Occupation	Whether your work is related to biotechnology? (0 = no; 1 = yes)	0.079 (0.271)	
Child	Whether your family has minors (≤15)? (0 = no; 1 = yes)	0.435 (0.496)	
Income	Monthly household disposable income (1 = RMB RMB 2001–4000; 3 = RMB 4001–6000; 4 = RMB 6001–8000; 5 = RMB 8001–10,000; 6 = RMB 10,001–12,000; 7 = RMB 12,000 or above)	3.398 (1.537)	

^a^ Standard deviation in the parentheses. ^b^ The population includes urban and rural residents based on information of 31 provinces from “2017 China Statistical Yearbook”. ^b^ RMB 6.80 = USD 1.

**Table 3:** Definition and descriptive statistics of the variables used in mixed logit model.

Variable	Description	Nationwide Consumers (N^a^ = 14,472)	Eastern Consumers (N^a^ = 6516)	Central Consumers (N^a^ = 5076)	Western Consumers (N^a^ = 2880)
Stated intention	0 = unwilling to pay; 1 = willing to pay	0.624 (0.484)	0.674 (0.469)	0.589 (0.492)	0.575 (0.494)
Whether to choose the option	0 = no; 1 = yes	0.333	0.333	0.333	0.333
		(0.471)	(0.471)	(0.471)	(0.471)
Allergen presence labelling	0 = no; 1 = yes; −1 = I do not choose either option A or B	−0.087	−0.095	−0.082	−0.076
		(0.757)	(0.751)	(0.760)	(0.765)
Nutrient and compositional change labelling		−0.044	−0.084	−0.023	0.007
		(0.788)	(0.759)	(0.802)	(0.821)
Traceability codes		−0.111	−0.111	−0.111	−0.111
		(0.737)	(0.737)	(0.737)	(0.737)
Price	RMB 46; RMB 53; RMB 60; 0 if option C is chosen	35.210	35.370	35.120	34.990
		(25.360)	(25.480)	(25.290)	(25.200)

^a^ N means the number of options. Standard errors in the parentheses.

**Table 4:** Estimation results of the mixed logit model.

	Nationwide Consumers		Eastern Consumers		Central Consumers		Western Consumers	
	All Samples	Stated Intention = 1	All Samples	Stated Intention = 1	All Samples	Stated Intention = 1	All Samples	Stated Intention = 1
Allergen presence labelling	0.427 ***	1.074 ***	0.725 ***	1.359 ***	0.286	0.994 ***	0.115	0.528 ***
	(0.112)	(0.081)	(0.170)	(0.130)	(0.189)	(0.135)	(0.237)	(0.172)
Nutrient and compositional change labelling	0.104	0.502 ***	0.530 ***	0.754 ***	−0.181	0.404 ***	−0.242	0.015
	(0.101)	(0.067)	(0.138)	(0.103)	(0.187)	(0.121)	(0.230)	(0.145)
Traceability codes	0.579 ***	1.118 ***	0.919 ***	1.382 ***	0.411 **	1.010 ***	−0.094	0.744 ***
	(0.115)	(0.092)	(0.166)	(0.142)	(0.202)	(0.156)	(0.320)	(0.213)
Price	−0.065 ***	−0.061 ***	−0.082 ***	−0.079 ***	−0.055 ***	−0.054 ***	−0.040 ***	−0.030 ***
	(0.005)	(0.005)	(0.007)	(0.007)	(0.008)	(0.008)	(0.011)	(0.010)
Coefficient covariance								
v11	1.314 ***	0.682 ***	1.471 ***	0.893 ***	−1.230 ***	0.502 ***	0.988 ***	0.591 ***
	(0.111)	(0.085)	(0.172)	(0.125)	(0.175)	(0.111)	(0.201)	(0.133)
v 21	0.696 ***	0.200	0.419 **	−0.093	−1.055 ***	0.210	0.815 ***	0.688 ***
	(0.127)	(0.140)	(0.207)	(0.179)	(0.219)	(0.242)	(0.251)	(0.140)
v 31	0.852 ***	0.194	0.914 ***	0.366 **	−0.999 ***	0.499 **	0.336	−0.958 ***
	(0.120)	(0.121)	(0.157)	(0.145)	(0.208)	(0.194)	(0.308)	(0.269)
v 22	1.031 ***	0.911 ***	1.054 ***	0.983 ***	1.029 ***	1.055 ***	−0.410	−0.032
	(0.096)	(0.095)	(0.173)	(0.133)	(0.172)	(0.148)	(0.272)	(0.379)
v 32	−0.574 ***	−0.666 ***	−0.509 ***	−0.470 ***	−0.992 ***	−0.804 ***	0.944 **	−0.584
	(0.117)	(0.115)	(0.192)	(0.141)	(0.163)	(0.168)	(0.369)	(0.470)
v 33	0.984 ***	0.683 ***	0.865 ***	0.671 ***	0.406	0.053	1.655 ***	0.758 **
	(0.130)	(0.124)	(0.172)	(0.142)	(0.287)	(0.422)	(0.289)	(0.303)
Log likelihood	−5118.33	−4397.01	−2353.36	−2054.82	−1741.35	−1477.03	−990.21	−831.17
LR chi2(6)	3858.12 ***	835.34 ***	1739.81 ***	464.86 ***	1352.73 ***	241.50 ***	717.31 ***	135.14 ***
*P* value	<0.001	<0.001	<0.001	<0.001	<0.001	<0.001	<0.001	<0.001
Number of options	14472	9036	6516	4392	5076	2988	2880	1656

The parameters in the bottom panel of the output are the elements of the lower-triangular matrix L. Standard errors in the parentheses. *** and ** denote statistical significance at the 0.01, 0.05 and 0.10 levels, respectively.

**Table 5:** Willingness to pay for a premium for enhanced mandatory labeling information of the GM soybean oil.

Attribute	Nationwide Consumers		Eastern Consumers		Central Consumers		Western Consumers	
	All Samples	Stated Intention = 1	All Samples	Stated Intention = 1	All Samples	Stated Intention = 1	All Samples	Stated Intention = 1
Allergen presence labelling	6.57 ***	17.50 ***	8.87 ***	17.23 ***	5.15	18.28 ***	2.85	17.58 ***
	(3.72, 9.42)	(15.85, 19.15)	(5.66, 12.09)	(15.18, 19.28)	(−0.59, 10.89)	(15.17, 21.38)	(−7.82, 13.52)	(10.12, 25.03)
Nutrient and compositional change labelling	1.60	8.17 ***	6.48 ***	9.56 ***	−3.27	7.42 ***	−5.99	0.51
	(−1.34, 4.54)	(6.35, 10.00)	(3.42, 9.55)	(7.20, 11.93)	(−10.36, 3.83)	(3.73, 11.10)	(−18.96, 6.98)	(−8.73, 9.76)
Traceability codes	8.92 ***	18.22 ***	11.24 ***	17.52 ***	7.41 ***	18.57 ***	−2.33	24.76 ***
	(5.98, 11.86)	(16.34, 20.09)	(8.20, 14.28)	(15.53, 19.52)	(1.17, 13.63)	(14.88, 22.26)	(−18.37, 13.71)	(11.91, 37.61)

Lower bound and upper bound for 95% confidence interval in the parentheses. *** denote statistical significance at the 0.01, 0.05 and 0.10 levels, respectively.

## Data Availability

Not applicable.

## Supplementary Materials

The following are available online at https://www.mdpi.com/article/10.3390/foods10040736/s1, Table S1. Prices of the “X” brand edible oil in the same size.
