# Supplementary material for: Willingness to Pay for Enhanced Mandatory Labelling of Genetically Modified Soybean Oil: Evidence from a Choice Experiment in China
Source: Foods. 2021 Mar 30;10(4):736. doi: 10.3390/foods10040736 (PMC8067038; doi:10.3390/foods10040736)
Supplement: Supplementary file 1 [file foods-10-00736-s001.pdf]

Table S1. Prices of the "X" brand edible oil in the same size.

Species and Capacity Edible Oil	Price (RMB)
5L GM soybean oil	45.80
5L non-GM soybean oil	66.80
5L corn oil (non-GM)	78.00
5L peanut oil (non-GM)	128.50
5L rapeseed oil (non-GM)	59.90
5L sunflower oil (non-GM)	68.80
5L canola oil (non-GM)	65.80
5L sunflower oil blend (non-GM sunflower seed and GM soybean oil)	49.90
5L edible corn oil blend (non-GM corn and GM soybean oil)	49.90

The brand only has 4L peanut oil (non-GM) with the price of RMB 102.8. As the marginal cost of the package from 4–5L is small, we use a simple average to calculate the price of 5L peanut oil. The data are based on the price of "X" brand edible oil on Feb 17, 2016 at Suning (https://www.suning.com/). RMB 6.8 = USD 1.
